# Cancer-related mortality among solid organ transplant recipients: systematic review and meta-analysis

**DOI:** 10.3389/frtra.2026.1851859

**Published:** 2026-06-19

**Authors:** Charlotte Stephens, Rafeea Shah, Azm Hussain, Joseph Sturman, Yimeng Zhang, Felicity Baldwin, David Winter, Mike Hawkins, Raoul Reulen, Adnan Sharif

**Affiliations:** 1 Department of Nephrology and Transplantation, University Hospital Birmingham, Birmingham, United Kingdom; 2 Institute of Immunology and Immunotherapy, University of Birmingham, Birmingham, United Kingdom; 3 University Hospital Coventry and Warwickshire, Coventry, United Kingdom; 4 Applied Health Sciences, University of Birmingham, Birmingham, United Kingdom

**Keywords:** cancer, epidemiology, malignancy, meta-analysis, mortality, systematic review, transplantation

## Abstract

**Background:**

Organ transplantation is associated with an increased risk of cancer-related mortality; however, estimates from international cohorts vary widely. The aim of this systematic review and meta-analysis is to quantify this risk.

**Methods:**

We searched MEDLINE and OVID Embase and the Cochrane Central Register of Controlled Trials (CENTRAL) for population-based cohort studies reporting cancer-related mortality in adult solid organ transplant recipients up to 2nd October 2024. Meta-analysis was conducted using a restricted maximum likelihood random-effects model. Risk of bias was assessed using the Newcastle–Ottawa Scale.

**Results:**

Seventeen registry-based studies and multicentre cohort studies met inclusion criteria. The pooled standardised mortality ratio for cancer mortality was 2.21 (95% CI: 1.82–2.68). Meta-regression showed higher mortality after heart [mortality rate ratio (MRR) 2.08, 95% CI: 1.28–3.41] and lung (MRR: 1.83, 95% CI: 1.09–3.06; *p* = 0.025) transplantation compared with kidney transplantation. Geographic variation was observed, with lower mortality in East Asia.

**Conclusion:**

This meta-analysis demonstrates that solid organ transplantation is associated with an increased in overall cancer-related mortality compared with the general population, with variation by organ type and cancer site. Prostate cancer showed no excess mortality risk, supporting current guidance against additional screening. In contrast, mortality from non-Hodgkin's lymphoma was increased fivefold, consistent with the established link between immunosuppression and post-transplant lymphoproliferative disease. Notably, geographic variation was observed, with lower mortality in East Asia, likely reflecting differences lifestyle risk factors and health care structures. However significant between study heterogeneity exists, underscoring the need for tailored prevention strategies, rather than relying on aggregated global estimates in this high-risk population.

**Systematic Review Registration:**

https://www.crd.york.ac.uk/PROSPERO/view/CRD42024617474, identifier CRD42024617474.

## Introduction

Transplantation is a well-established, lifesaving treatment for organ failure. However, it's associated with an increased risk of malignancy, largely due to the requirement of lifelong immunosuppression and the heightened susceptibility of oncogenic viruses (1). With improved graft survival post-transplant and advances in clinical management, cancer is becoming a leading cause of mortality among transplant recipients. Prior to undergoing transplantation, patients are routinely counselled about this risk and cancer remains one of their greatest self-reported concerns (2).

Several studies have examined cancer-related mortality after solid organ transplantation. Most report an increased risk compared to the general population; however, the magnitude of risk is variable. Murray et al. (3) found a modest increase among kidney transplant recipients in Ireland with a standardised mortality ratio (SMR) of 1.35 [95% confidence intervals (CI): 1.17–1.62]. In contrast, Rosales et al. (4) reported a substantially higher SMR of 2.9 (95% CI: 2.7–3.1) for cancer-related death among all solid organ transplant recipients in Australia and New Zealand. When analyses are stratified by transplant types and individual cancer sites, the variation in reported mortality risk becomes even more difficult to interpret. The example of lung cancer, the leading cause of cancer-related death worldwide (5), further illustrates this heterogeneity. For example, Jeong et al. (6) found no increase in lung cancer mortality amongst kidney transplant recipients in South Korea (SMR: 0.7; 95% CI: 0.4–1.0) whereas data from Australia and New Zealand (4) demonstrated more than a two-and-a-half fold increased risk (SMR: 2.7; 95% CI: 2.3–3.2). Another example is colorectal cancer, where a recent systematic review in kidney transplant recipients demonstrated a modest increase in incidence and mortality, although substantial heterogeneity existed between included studies (7). Such discrepancies between studies likely reflect differences in population demographics, burden of comorbidities, and health care structures.

However this variability poses a challenge when attempting to translate published literature to real-world clinical practice. The heterogeneous data prohibits informed counselling for solid organ transplant candidates and hinders the development of evidence-based cancer screening programmes. Early detection of malignancy in solid organ transplant recipients is particularly important, as these patients often present with more advanced disease and demonstrate poorer responses to treatment (8), making effective screening strategies critical for improving mortality outcomes. However, cancer screening practices in the general population differ even among high-income countries, and in the absence of robust transplant-specific evidence, these population-based screening frameworks frequently inform post-transplant surveillance protocols (8). Consequently, variation in national screening policies further contribute to differences in reported cancer incidence and mortality across geographical settings.

To address the challenge this heterogenous data presents, the aim of our study was to conduct a systematic review and meta-analysis of existing cohort studies to provide a clearer understanding of cancer-related mortality following solid organ transplantation.

## Methods

The systematic review and meta-analysis was performed in line with the PRISMA (Preferred Reporting Items for Systematic reviews and Meta-Analyses) statement (9) and Meta-analysis Of Observational Studies in Epidemiology (MOOSE) guidelines (10) (Supplementary Table S1) and was prospectively registered with the international systematic review registry PROSPERO (CRD42024617474).

### Literature search

We searched MEDLINE and OVID Embase and Cochrane register for controlled trials (CENTRAL) databases for relevant studies, as well as backward/forward citation searching and reviewing grey literature. Searches were conducted from study inception until 2nd October 2024, with no language restrictions. Search strategies combined controlled vocabulary [e.g., MeSH (Medical Subject Heading) terms and Emtree], and free-text terms, using accepted truncation and wildcard searches relevant to each database (Supplementary Table S2).

### Eligibility criteria

We included all population cohort studies reporting cancer-related mortality in adult (≥18 years) solid organ transplant recipients. Eligible studies enrolled recipients with at least one type of site-specific solid organ transplant (e.g., kidney, liver, heart, lung) and reported cancer-specific mortality, defined as death attributed to a site-specific *de novo* cancer where specified by the study authors. We excluded single-centre studies to ensure that pooled estimates were robust, reflective of real-world practices and less susceptible to selection and publication bias. We also excluded studies limited to paediatric populations, those reporting exclusively on cancer recurrence or donor-derived cancers and studies of non-solid organ transplantation (e.g., haematopoietic stem cell transplants). Where multiple publications drew on the same underlying cohort (e.g., from the same registry, region, or study period), we retained the most contemporary or the most comprehensive report to avoid double counting.

### Study selection

Citations were imported into Covidence systematic review software (11) for screening. Duplicates were first removed automatically by Covidence, defined as records sharing the same title, year, volume and author. If remaining duplicates were found during review they were removed manually.

Title/abstract screening and subsequent full-text assessment were conducted independently by two reviewers; CS screened all records at both stages with RS, AH, FB, YZ, and JS acting as a second reviewer for each record. The researchers were blinded to each other's decisions. If a consensus regarding a study couldn't be reached through discussion, AS served as a third reviewer for final adjudication. For studies not reported in English, we sought full-text translations electronically and via the University of Birmingham library. If a translation could not be obtained, the study was excluded. For reports without accessible abstracts or full texts, we attempted retrieval via the interlibrary loan system and by contacting the corresponding authors.

### Data extraction

Data was extracted into a pre-piloted spreadsheet (Excel, Microsoft Corp, WA, USA), that also included an assessment of risk of bias. Extracted items included study characteristics (design, geographical area, inclusion/exclusion criteria), population characteristics (age, sex, ethnicity), transplant information (types and numbers), cancer information [site(s), time from transplant to diagnosis], and mortality outcomes. Scoping searches revealed cancer-related mortality was reported using heterogenous metrics, therefore all available mortality assessments were recorded, including standardised mortality ratios (SMRs), adjusted hazard ratios (HR), crude mortality rates (CMR) and percentage survival at prespecified end points. SMRs compared observed mortality in transplant recipients with expected mortality in the age-, sex-, and calendar year-matched general population. Hazard ratios represented adjusted within-study relative risks of a cohort that included both transplant and non-transplant recipients, while crude mortality rates described absolute mortality within the transplant population without external population comparison.

### Risk of bias

Risk of bias was assessed during data collection using the Newcastle-Ottawa Scale (NOS) for comparative non-randomised cohort studies (12). The NOS evaluates three domains: Selection, Compatibility and Outcome, with a maximum score of 9 stars across the sections. Although no formal cut-offs exist, general consensus accepts that ≥7 stars represents a low risk of bias (13). We therefore defined our assessment of bias with ≥7 stars as low risk of bias (high quality study), 6 stars as moderate risk and ≤5 stars to be high risk of bias (low quality study).

### Data synthesis and analysis

We conducted a meta-analysis using a restricted maximum likelihood (REML) random-effects model, anticipating substantial between-study heterogeneity in line with recommendations from the Cochrane Statistical Methods Group (14). Where available, we used SMRs to compare cancer mortality among solid organ transplant recipients with that of the general population. All relative effect measures were log-transformed, pooled using inverse-variance weighting, and reported with 95% confidence intervals.

When SMRs were not reported, we instead used CMRs. If CMRs were missing, we estimated them as deaths divided by person-years at risk. Where person-years were not reported, we approximated person-years using the average follow-up and the number of study patients.

Between study heterogeneity was assessed visually using forest plots and quantified using the *I*^2^ statistic. The *I*^2^ statistic roughly quantifies the level of heterogeneity with ≤40% indicating low/unimportant, 30%–60% indicating moderate, 50%–90% indicating substantial, and >90% indicating considerable levels of heterogeneity (14). Analyses were stratified by transplanted organ and by cancer site. To explore sources of heterogeneity, we performed study-level meta-regression, applying Knapp-Hartung standard error adjustment given the small number of studies and high level of heterogeneity (15, 16).

Publication bias (small-study effects) was assessed visually with funnel plots and with Egger's regression test (17). All analyses were conducted using Stata/SE 19.5 (StataCorp, College Station, TX).

## Results

### Study selection and characteristics

**Figure 1:**
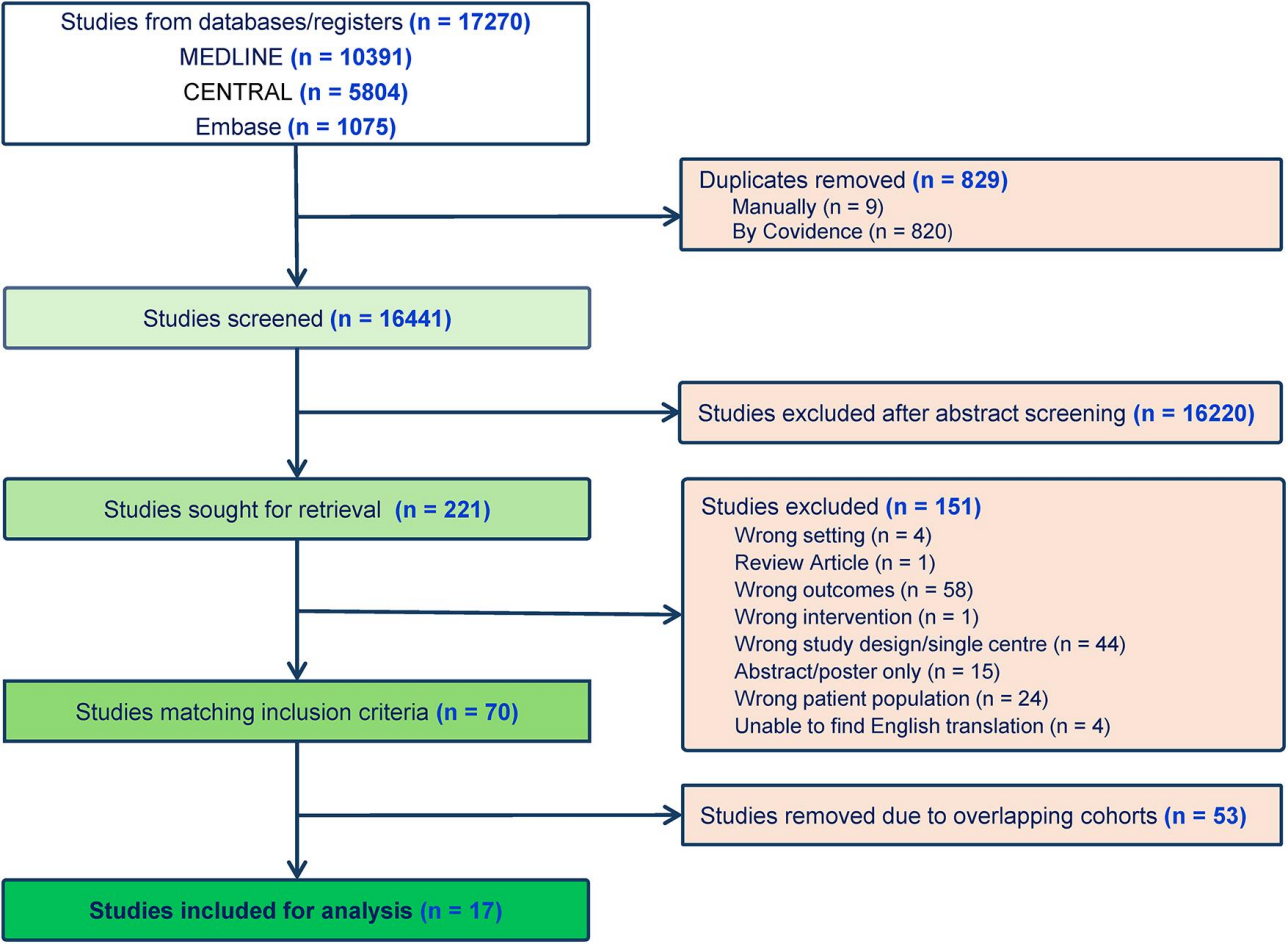
PRISMA (preferred reporting items for systematic reviews and meta-analyses) flow diagram for systematic review and meta-analysis of cancer related mortality after solid organ transplantation.

Following the removal of duplicates, 16,441 studies were screened of which 16,220 were excluded after title or abstract review. From the 221 studies reviewed during the full text screening process, 70 met the inclusion criteria. After excluding a further 53 studies due to overlapping cohorts or registries, 17 studies were included in the final systematic review and meta-analysis (3, 4, 6, 18–31). Figure 1 shows a PRISMA flow diagram detailing the study selection.

All the studies were retrospective in design. From the 17 studies, 12 were national or regional registry-based studies and the remaining 5 were multicentre cohort studies (minimum of three hospitals). Four studies reported on all major solid organ graft types (kidney, liver, heart and lung), 8 focussed on kidney transplants alone, 3 on liver, 1 on heart and 1 on lung. All studies reported cancer-related mortality across multiple cancer sites (see Supplementary Table S3 for summary demographics of included studies).

The included studies encompassed a total of 782,053 transplant recipients, although one study (22) restricted analysis to only those who had developed cancer. There were 17,975 recorded cancer deaths in total. Study sample sizes ranged from 179 to 671,127 participants and recruitment periods spanned from 1965 to 2020. In total, 9 of the studies were conducted in Europe, 2 in North America, 5 in East Asia, and 1 in Australia and New Zealand. Among the 12 studies that reported sex distribution, 62.4% of participants were male (480,003/769,038).

### Study quality

All 17 studies achieved a Newcastle–Ottawa Scale score between 7 and 9, indicating low risk of bias, with a mean score of 7.2. The most missed points related to incomplete reporting of loss to follow-up and limited demographic detail (Supplementary Table S4).

### Systematic review of evidence

10 of the 17 studies (58%) directly compared cancer-related mortality in transplant recipients with the general population. Of these, 9 (90%) reported an increase in mortality. Jackson-Spence et al. (23) was the single exception, although the study acknowledged the limitations of their hospital administration dataset. SMRs ranged from 0.75 (95% CI: 0.71–0.79) to 2.9 (95% CI: 2.7–3.1).

### Meta-analysis of cancer mortality by transplant type

**Figure 2:**
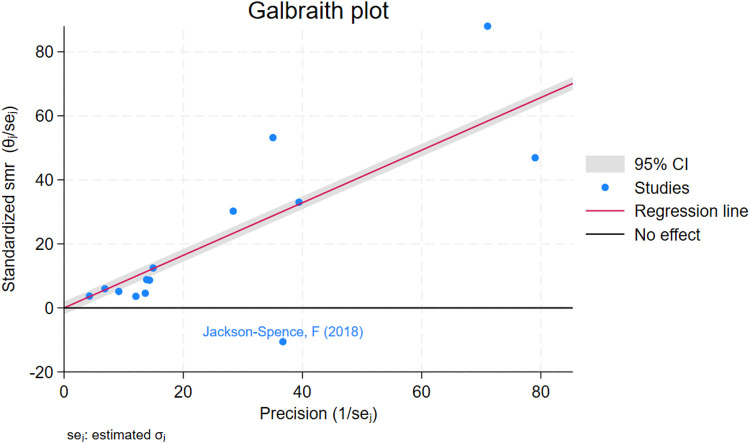
Galbraith plot of the pooled risk ratios for cancer mortality after solid organ transplantation.

**Figure 3:**
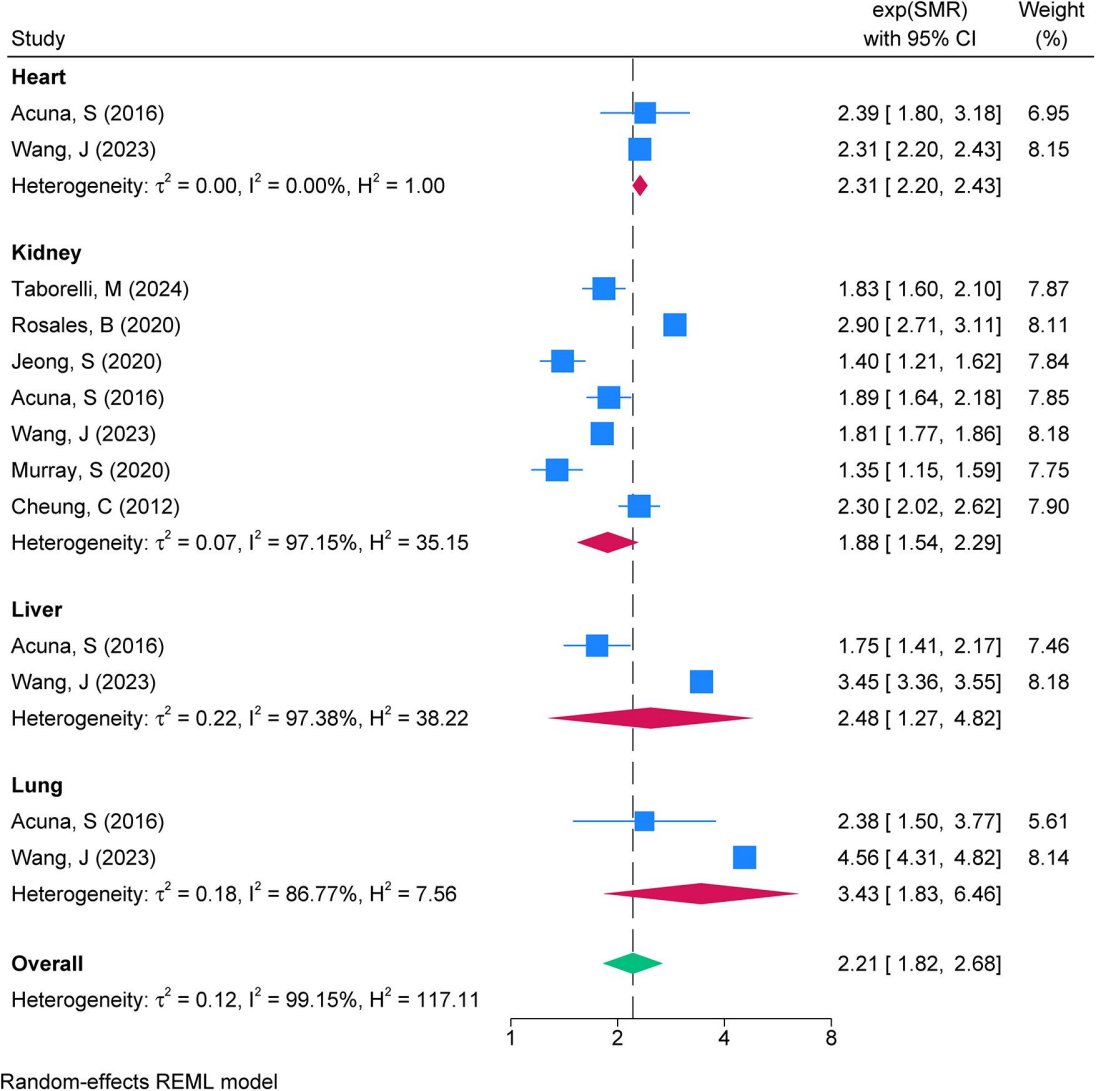
Forest plot of pooled risk ratios for cancer-related mortality compared with the general population, stratified by transplant type. The small blue boxes represent individual studies, with horizontal whiskers denoting 95% confidence intervals. The large red diamonds indicate the summary effect size within each transplant type, with the centre marking the pooled estimate and the width showing the 95% confidence interval. The large green diamond depicts the overall pooled risk ratio, with its centre corresponding to the combined estimate and its width to the 95% confidence interval. REML, restricted maximum likelihood.

Eight studies (3, 4, 6, 18, 23, 24, 30, 32) reported SMRs, allowing for analysis stratified by transplant type. However, the study by Jackson-Spece et al. was excluded due to the self-reported data limitations and because it was identified as a significant outlier in the Galbraith plot (Figure 2). The overall pooled SMR from the remaining seven studies was 2.21 (95% CI: 1.82–2.68) (Figure 3), indicating a more than two-fold increase in cancer-related mortality among transplant recipients. Between-study heterogeneity was substantial (*I*^2^ = 99.2%). Stratified analysis looking at transplanted organ type demonstrated lung transplant recipients had the highest cancer-related mortality risk (3.43; 95% CI: 1.83–6.46), followed by liver (2.48; 95% CI: 1.27–4.82) and heart (2.31; 95% CI: 2.2–2.43), with kidney showing the lowest estimate (1.88; 95% CI: 1.54–2.29).

### Meta-analysis and meta-regression of cancer mortality by transplant type

**Figure 4:**
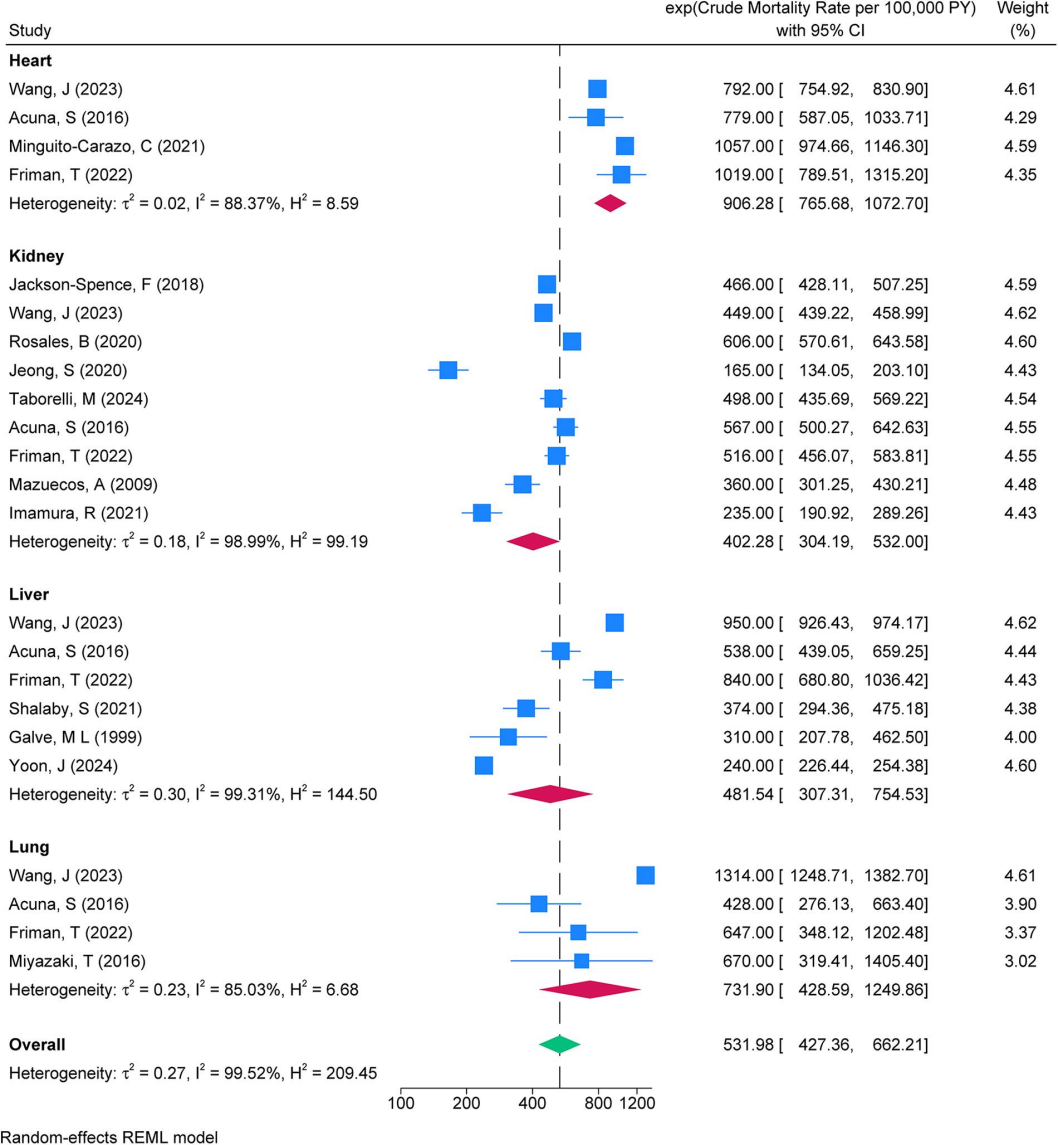
Forest plot of crude mortality rates for cancer-related mortality, stratified by transplant type. The small blue boxes represent individual studies, with horizontal whiskers denoting 95% confidence intervals. The large red diamonds indicate the summary effect size within each transplant type, with the centre marking the pooled estimate and the width showing the 95% confidence interval. The large green diamond depicts the overall pooled crude mortality rate, with its centre corresponding to the combined estimate and its width to the 95% confidence interval. The dashed vertical line marks the overall pooled crude mortality rate for reference. REML, restricted maximum likelihood.

Fifteen studies (4, 6, 18–21, 23–30, 32) reported or allowed calculation of crude mortality rates (CMRs). The pooled CMR across all organs was 531.98 per 100,000 person-years (95% CI: 427.4–662.2) as shown in Figure 4. As four studies required approximation of person-years at risk, a sensitivity analysis excluding these studies was performed. The pooled CMR after exclusion was 486.34 per 100,000 person-years (95% CI: 338.91–679.92), which was broadly consistent with the primary analysis, suggesting that inclusion of studies did not significantly introduce systematic bias.

Stratified by transplant type, pooled CMRs were 402.3 for kidney (95% CI: 304.2–532.0), 906.3 for heart (95% CI: 765.7–1,072.7), 481.5 for liver (95% CI: 307.3–754.5), and 731.9 for lung (95% CI: 428.6–1,249.9). Between-study heterogeneity was substantial overall (*I*^2^ = 99%).

Meta-regression adjusting for transplant type and geographical area, demonstrated that heart [Mortality rate ratio (MRR) 1.94, 95% CI: 1.23–3.05; *p* = 0.007] and lung (MRR 1.83, 95% CI: 1.09–3.06; *p* = 0.025) transplantation were associated with higher cancer mortality relative to kidney transplantation (Table 1). Liver transplantation (MRR: 1.21, 95% CI: 0.82–1.79; *p* = 0.317), was not significantly different. Compared with Europe, cancer mortality rates did not differ in North America (MRR: 1.1, 95% CI: 0.77–1.57; *p* = 0.60), or Australia and New Zealand (MRR: 1.36, 95% CI: 0.64–2.9; *p* = 0.403). However, they were significantly lower in East Asia (MRR: 0.48, 95% CI: 0.3–0.76; *p* = 0.004). The model explained 63% of the between-study variability (*R*^2^ = 63.49%), although residual heterogeneity remained high (*I*^2^ = 95.8%).

**Table 1:** Meta-regression for cancer mortality.

Group	MRR (95% CI)	*p* value
Transplanted organ		
Kidney	1*	
Heart	1.94 (1.23–3.05)	0.007
Lung	1.83 (1.09–3.06)	0.025
Liver	1.21 (0.82–1.79)	0.317
Geographical region		
Europe	1*	
North America	1.1 (0.77–1.57)	0.596
East Asia	0.48 (0.30–0.76)	0.004
Australia and New Zealand	1.36 (0.64–2.89)	0.403

MRR, mortality rate ratio; CI, confidence interval.

Meta-regression results for cancer mortality based on crude mortality rates, adjusted for transplanted organ and geographical region.

* Reference category.

In a separate model adjusting for age, sex, transplant type, and geography (*n* = 9 studies), neither age nor sex was significantly associated with cancer mortality.

### Subgroup analysis by cancer site

Nine studies (3, 4, 6, 18, 23–25, 30, 32) reported mortality outcomes allowing for sub-group analysis for site-specific cancers [colorectal, lung, breast, prostate and non-Hodgkin's lymphoma (NHL)], however the study by Jackson-Spence et al. was excluded due to the issues previously described. The summary of pooled results are presented in Table 2.

**Table 2:** Pooled SMRs for site specific cancers among solid organ transplant recipients.

Cancer site	Pooled SMRs (95% CI)	*I*^2^ (%)
Colorectal	1.8 (1.3–2.48)	90
Lung	1.6 (1.24–2.06)	92.8
Breast	1.16 (0.82–1.63)	76.1
Prostate	0.94 (0.69–1.28)	63
Non-Hodgkin Lymphoma	5.65 (2.85–11.23)	99.1

SMR, standardised mortality ratio.

Pooled SMRscfor each cancer type.

NHL showed the highest excess risk, with a pooled SMR of 5.65 (95% CI: 2.85–11.23), corresponding to more than a five-fold increase in mortality compared with the general population. Colorectal cancer (SMR 1.8, 95% CI: 1.3–2.48) and lung cancer (SMR: 1.6, 95% CI: 1.24–2.06) demonstrated more modest but statistically significant increases in mortality risk. In contrast, breast cancer (SMR: 1.16, 95% CI: 0.82–1.63) and prostate cancer (SMR: 0.94, 95% CI: 0.69–1.28) were not associated with excess mortality following transplantation.

Skin cancer mortality was reported in 10 included studies; however, two studies reported no skin cancer-related deaths, while the remaining studies reported heterogeneous outcome measures that did not permit pooled quantitative analysis. Consequently, skin cancer mortality was not included in the meta-analysis. Nevertheless, we acknowledge that skin cancers represent one of the highest-incidence malignancies following solid organ transplantation (33, 34), despite often contributing proportionally less to overall cancer-related mortality.

Six studies (3, 4, 6, 18, 24, 30) allowed for subgroup analysis of specific cancers among kidney transplant recipients (Supplementary Table S5 for pooled results). However, equivalent analyses could not be performed for other solid organ transplant types because of insufficient study numbers presented SMRs. Broadly similar patterns were observed among kidney transplant recipients compared with the overall cohort, with NHL demonstrating the highest pooled SMR at 7.62 (95% CI: 3.30–17.59), while prostate cancer showed no significant excess mortality risk compared with the general population (SMR: 1.09; 95% CI: 0.71–1.69). In contrast, breast cancer mortality was significantly increased among kidney transplant recipients (SMR: 1.54; 95% CI: 1.14–2.07), a finding not observed in the overall pooled cohort.

### Publication bias

The funnel plot appeared symmetrical (Supplementary Figure 1), which was confirmed with an Egger's regression test of *β*_1_ = 0.0, *p* = 0.09, suggesting no evidence of small-study effects or publication bias.

## Discussion

### Findings

This systematic review and meta-analysis of 782,053 transplant recipients demonstrates that solid organ transplantation is associated with an increased risk of cancer related mortality compared to the general population. The pooled SMR across included studies was 2.21 (95% CI: 1.82–2.68), indicating a more than 2-fold increase in cancer mortality. Supporting this observation, the pooled CMR was 531.98 per 100,000 person years; 2-3 times higher than the national rates reported in the UK (251.6) (35), and South Korea (158.2) (36). Although the increased risk was consistent across nearly all studies and analyses, substantial heterogeneity remained despite subgroup and meta-regression approaches, highlighting the complexity of interpreting cancer outcomes in this population.

The increased cancer mortality was evident across all transplant types, though the magnitude varied. Heart and lung transplant recipients experienced significantly higher mortality compared with kidney recipients, whereas liver transplants demonstrated a similar risk. This difference likely reflects the higher level of immunosuppression required for cardiothoracic transplantation, leading to the documented high incidence of cancer among heart and lung transplant recipients (37, 38). Hearts and lungs are considered “tolerance-resistant,” meaning they lack immunological properties that promote long-term acceptance of the graft, and are therefore more prone to rejection (39). By contrast, liver allografts are relatively “tolerance-prone,” eliciting weaker rejection responses, and require less immunosuppression (40).

Compared with Europe, cancer mortality rates were similar in North America (MRR 1.1, *p* = 0.6) and Australia/New Zealand (MRR: 1.36, *p* = 0.4), whereas significantly lower rates were observed in East Asia (Japan, South Korea, and Hong Kong; MRR: 0.48, *p* = 0.004). These findings align with prior evidence demonstrating that Japan has the lowest cancer mortality and longest life expectancy among G7 nations (41). Notably, despite lower per capita cancer expenditure, Japan and South Korea report significantly lower cancer mortality than the United States (42), suggesting that population-level risk factors may be more influential than healthcare spending. Lower prevalence of obesity may contribute; in 2022, obesity affected only 5.6% of the Japanese population and 7.3% in South Korea, compared with 42.7% in the United States and 34.2% in New Zealand (43). Smoking prevalence is also comparatively lower in Japan and South Korea than in many Western countries (44). Coupled with a healthier diet (41), these factors may provide some explanation for the lower cancer related mortality observed in East Asia. In addition to differing risk factor profiles, the healthcare systems in Japan and South Korea are considered among the highest performing globally, characterised by high levels of efficiency and effectiveness alongside near-universal population coverage and relatively low insurance premiums with most medical treatments broadly funded (45, 46). Finally, Japan operates a relatively intensive national cancer screening programme compared to other countries, including cervical cancer screening every two years from age 20, and gastric, breast, lung, and colorectal cancer screening from age 40 (47), which may facilitate earlier detection of malignancies, earlier stage at diagnosis, and improved overall prognosis.

Subgroup analysis by cancer site showed marked variation in risk. NHL carried the highest relative mortality, likely reflecting the known increased incidence of NHL following solid organ transplantation due to the strong association between immunosuppression-related impaired immune surveillance and Epstein–Barr Virus driven post-transplant lymphoproliferative disease (48).

By contrast, no increased mortality risk was observed for breast and prostate cancer. For prostate cancer, the lower between-study heterogeneity suggests a more consistent lack of increased mortality risk, indicating that some current screening guidelines may be unnecessarily burdensome. The American Society of Transplant (AST) guidelines (49) recommend annual prostate-specific antigen testing and digital rectal examinations for men over 50 who have a life expectancy of greater than 10 years. Our findings support the more conservative Kidney Disease Improving Global Outcomes (KDIGO) (50) guidelines which do not recommend additional prostate cancer screening in transplant recipients. According to the Global Cancer Observatory, cancer deaths worldwide are expected to nearly double between 2022 and 2050, rising from 9.7 million to 18.1 million annually (51). In parallel, the cumulative cohort of transplantation recipients is also expanding: solid organ transplant procedures have increased by 52% between 2010 and 2022 (52). These trends intersect with the rising prevalence of underlying conditions leading to end organ failure. Metabolic dysfunction-associated steatotic liver disease (MASLD), the leading indication for liver transplantation in the US (53), is projected to affect more than 40% of Americans by 2050 (54). Chronic kidney disease (stages 3–5) is expected to affect more than 10% of the populations of North and Central Latin America, Eastern Europe, North and Sub-Saharan Africa, the Middle East, Central Asia and high-income Asia Pacific over the same time period (55). Taken together, these projections suggest cancer mortality after transplantation will become an increasingly pressing challenge.

Optimal clinical management of this growing burden of post-transplant cancer has yet to be defined, with no dedicated post-transplantation guidelines due to a lack of strong clinical evidence. While intensified screening processes may detect earlier cancers, this needs to be patient, cancer site and transplant type specific and balance efficacy with cost effectiveness. Screening must also be balanced with the potential harm through false positives, invasive investigations, overdiagnosis and patient anxiety. Moreover, observational data suggest that uptake of cancer screening among transplant recipients is low (56) with lower adherence associated with socioeconomic disadvantage, lower health literacy and younger age (57). Many patients would prioritise allograft health over preventative cancer measures (58), despite outcomes often being worse than in the general population once cancer develops (59). As such, prevention should also be prioritised through minimisation of immunosuppression where feasible and risk modification such as smoking cessation and weight management. Rather than increased cancer screening frequency, solid organ transplant recipients should be counselled to adhere to their routinely invited cancer screening tests.

### Strengths and limitations

The strengths of this review include a large, pooled cohort of more than 782,000 transplant recipients, with exclusion of all overlapping cohorts and adherence to a prespecified, systematic review protocol. The quality of all included studies was high with low risk of bias as indicated by the Newcastle-Ottawa scale.

Several limitations must also be acknowledged. First, between-study heterogeneity remained high despite stratification by organ type, geography, and demographics. This likely reflects variation in immunosuppressive regimens, healthcare systems, population characteristics, cancer screening practice and comorbidity burdens; factors difficult to fully account for in a population-level analysis. As such, the pooled estimates should be interpreted with caution, as they may not be directly generalisable to specific clinical settings or patient populations and shouldn't be interpreted as individual-level risk estimates for clinical decision-making.

Second, our analysis focused on cancer mortality because it reflects the ultimate clinical burden of cancer in transplant recipients. However, mortality is a composite outcome, determined by both the elevated incidence of cancer (34) in this population and their poorer survival following diagnosis (59). This complexity should be considered when interpreting our findings.

Third, to fully inform cancer prevention strategies, future research should also assess cancer morbidity and health economic impact in addition to incidence and mortality.

Additionally, differences in national cancer screening programmes may also introduce lead-time bias, whereby cancers are diagnosed earlier without necessarily changing disease-specific outcomes. Variability in screening intensity and diagnostic practices may therefore have influenced observed survival and mortality estimates across geographical regions.

Finally, although we restricted inclusion to multicentre and population-based cohort studies to prioritise large-scale epidemiological estimates and improve generalisability, we acknowledge that this approach has limitations. Registry-based datasets frequently lack granular clinical information, including immunosuppressive regimens, adherence to cancer screening practices and detailed phenotypic characteristics, which are important determinants of post-transplant oncological risk. In contrast, single-centre studies may provide more detailed clinical characterisation and follow-up data, although often at the expense of broader population representativeness.

## Conclusion

In conclusion, solid organ transplantation is associated with a significant increase in overall cancer-related mortality compared with the general population, with variation by organ type, cancer site and geographical location. Some cancers showed no excess mortality risk, supporting current KDIGO guidelines that recommend no additional screening. However, other cancers such as PTLD demonstrated a significant risk for excess mortality. Future research should focus on the development and evaluation of transplant-specific cancer prevention and screening strategies, ideally through prospective trials, to support evidence-based care in this high-risk population. In addition, other factors that may impact upon excess cancer mortality risk for transplantation recipients (such as deviation in cancer management vs. the general population) is worthy of further investigation.

## Data Availability

The original contributions presented in the study are included in the article/Supplementary Material, further inquiries can be directed to the corresponding author.
