# Supplementary material for: Cancer-related mortality among solid organ transplant recipients: systematic review and meta-analysis
Source: Front Transplant. 2026 Jun 19;5:1851859. doi: 10.3389/frtra.2026.1851859 (PMC13328252; doi:10.3389/frtra.2026.1851859)
Supplement: Supplementary file 1 [file Table1.docx]

**Supplementary Material**

**Table S1 - MOOSE Checklist for Meta-analyses of Observational Studies**

| Item Number | Recommendation | Section |
| --- | --- | --- |
| Reporting of background should include | | |
| 1 | Problem definition | Introduction |
| 2 | Hypothesis statement | Introduction |
| 3 | Description of study outcome(s) | Introduction |
| 4 | Type of exposure or intervention used | Introduction |
| 5 | Type of study designs used | Methods |
| 6 | Study population | Methods |
| Reporting of search strategy should include | | |
| 7 | Qualifications of searchers (eg, librarians and investigators) | Methods |
| 8 | Search strategy, including time period included in the synthesis and key words | Methods & Supplementary Table S2 |
| 9 | Effort to include all available studies, including contact with authors | Methods |
| 10 | Databases and registries searched | Methods & Supplementary Table S2 |
| 11 | Search software used, name and version, including special features used (eg, explosion) | Methods |
| 12 | Use of hand searching (eg, reference lists of obtained articles) | Methods |
| 13 | List of citations located and those excluded, including justification | Methods & Figure 1 |
| 14 | Method of addressing articles published in languages other than English | Methods |
| 15 | Method of handling abstracts and unpublished studies | Methods |
| 16 | Description of any contact with authors | N/A |
| Reporting of methods should include | | |
| 17 | Description of relevance or appropriateness of studies assembled for assessing the hypothesis to be tested | Methods |
| 18 | Rationale for the selection and coding of data (eg, sound clinical principles or convenience) | Methods |
| 19 | Documentation of how data were classified and coded (eg, multiple raters, blinding and interrater reliability) | Methods |
| 20 | Assessment of confounding (eg, comparability of cases and controls in studies where appropriate) | Methods |
| 21 | Assessment of study quality, including blinding of quality assessors, stratification or regression on possible predictors of study results | Methods |
| 22 | Assessment of heterogeneity | Methods |
| 23 | Description of statistical methods (eg, complete description of fixed or random effects models, justification of whether the chosen models account for predictors of study results, dose-response models, or cumulative meta-analysis) in sufficient detail to be replicated | Methods |
| 24 | Provision of appropriate tables and graphics | Table 1-2, Figures 1-4 |
| Reporting of results should include | | |
| 25 | Graphic summarizing individual study estimates and overall estimate | Figures 3-4 |
| 26 | Table giving descriptive information for each study included | Supplementary Table S3 |
| 27 | Results of sensitivity testing (eg, subgroup analysis) | Results & Table 2 |
| 28 | Indication of statistical uncertainty of findings | Results & Table 2 |
| Reporting of discussion should include | | |
| 29 | Quantitative assessment of bias (eg, publication bias) | Results & Supplementary Figure S1 |
| 30 | Justification for exclusion (eg, exclusion of non-English language citations) | Figure 1 |
| 31 | Assessment of quality of included studies | Results & Supplementary Table S4 |
| Reporting of conclusions should include | | |
| 32 | Consideration of alternative explanations for observed results | Discussion |
| 33 | Generalization of the conclusions (ie, appropriate for the data presented and within the domain of the literature review) | Discussion |
| 34 | Guidelines for future research | Discussion |
| 35 | Disclosure of funding source | Front page |

**Table S2 – Search strategies from inception to 2^nd^ October 2024**

MEDLINE (OVID) search strategy

1. exp Neoplasms/
2. neoplas*.ti,ab,kw.
3. cancer*.ti,ab,kw.
4. (tumour* or tumor*).ti,ab,kw.
5. malignan*.ti,ab,kw.
6. (oncology or oncologic or oncologist*).ti,ab,kw.
7. metasta*.ti,ab,kw.
8. carcinoma*.ti,ab,kw.
9. adenocarcinoma*.ti,ab,kw.
10. 1 or 2 or 3 or 4 or 5 or 6 or 7 or 8 or 9
11. Exp Organ Transplantation/
12. exp Heart Transplantation/
13. Kidney Transplantation/
14. Liver Transplantation/
15. exp Lung Transplantation/
16. Pancreas Transplantation/
17. Organ transplant*.ti,ab,kw.
18. Heart transplant*.ti,ab,kw.
19. Kidney transplant*.ti,ab,kw.
20. Liver transplant*.ti,ab,kw.
21. Lung transplant*.ti,ab,kw
22. Pancreas transplant*.ti,ab,kw.
23. ((organ or heart or heart-lung or kidney or renal or liver or lung or pancreas) adj2 transplant*).ti,ab,kw.
24. exp Transplant recipients/
25. transplant recipient*.ti,ab,kw.
26. 11 or 12 or 13 or 14 or 15 or 16 or 17 or 18 or 19 or 20 or 21 or 22 or 23 or 24 or 25
27. exp Mortality/
28. death.ti,ab,kw.
29. mortality.ti,ab,kw.
30. exp Survival Analysis/
31. "survival analysis".ti,ab,kw.
32. “survival rate”.ti,ab,kw.
33. 27 or 28 or 29 or 30 or 31 or 32
34. 10 and 26 and 33

EMBASE Classic search strategy

1. exp neoplasm/
2. neoplas*.tw.
3. cancer*.tw.
4. (tumour* or tumor*).tw.
5. malignan*.tw.
6. (oncology or oncologic or oncologist*).tw.
7. metasta*.tw.
8. carcinoma*.tw.
9. adenocarcinoma*.tw.
10. 1 or 2 or 3 or 4 or 5 or 6 or 7 or 8 or 9 or 10
11. organ transplantation/
12. exp heart transplantation/
13. exp kidney transplantation/
14. exp liver transplantation/
15. exp lung transplantation/
16. exp pancreas transplantation/
17. ((organ or heart or heart-lung or kidney or renal or liver or lung or pancreas) adj2 transplant*).tw.
18. Exp transplant recipients/
19. 11 or 12 or 13 or 14 or 15 or 16 or 17 or 18
20. Mortality/
21. death/
22. death.ti,ab,kw.
23. mortality.ti,ab,kw.
24. Survival Analysis/
25. "survival analysis".ti,ab,kw.
26. 20 or 21 or 22 or 23 or 24 or 25
27. 10 and 19 and 26
28. Limit 27 to (editorial or letter)
29. 27 not 28
30. Limit 29 to humans
31. Limit 30 to multicenter study

Cochrane library search strategy

1. MeSH descriptor: [Cause of Death] this term only
2. MeSH descriptor: [Neoplasms] this term only
3. MeSH descriptor: [Organ Transplantation] explode all trees
4. MeSH descriptor: [Survival Analysis] explode all trees
5. (Neoplas* or cancer or tumour* or tumor or malignan*):ti,ab,kw
6. ("mortality" or survival analysis or survival rate or death):ti,ab,kw
7. (transplant* or organ transplant* or kidney transplant* or liver transplant* or heart transplant* or lung transplant* or pancreas transplant* or transplant recipient*):ti,ab,kw
8. MeSH descriptor: [Survival Rate] this term only
9. MeSH descriptor: [Mortality] this term only
10. #1 or #4 or #6 or #8 or #9
11. #2 or #5
12. #3 or #7
13. #10 and #11 and #12

**Table S3: Summary Characteristics of Included Studies**

| **Authors** | **Year** | **Country** | **Recruitment  period** | **Cohort type^a^ (N)** | **Transplanted  organ** | **Transplanted  cohort** | **Male (%)** | **Age^b^ (Years)** | **Effect measure** |
| --- | --- | --- | --- | --- | --- | --- | --- | --- | --- |
| Murray et al. | 2020 | Ireland | 1994-2014 | Registry | Kidney | 3,267 | 70.8^c^ | 56.0 ^‡ c d^ | HRs, SMRs and 5-, 10-, 15- year mortality rate |
| Taborelli  et al. | 2024 | Italy | 2003-2020 | Multi-centre (17) | Kidney | 7,373 | 63.6 | 50.5^d^ | SMRs |
| Shalaby et al. | 2021 | Italy | 1985-2014 | Multi-centre (9) | Liver | 2,653 | 74.9^d^ | 53 ^‡ d^ | 5-, 10- year mortality rate |
| Yoon et al. | 2024 | South Korea | 2006-2017 | Registry | Liver | 10,338 | 72.5 | 52.8 | 1- and 5- year mortality rate,  CMR per 1000 PY |
| Imamura et al. | 2021 | Japan | 1965-2016 | Multi-centre (3) | Kidney | 1,973 | 61.7^d^ | 39^d^ | 5-, 10-, 20- year mortality rate |
| Rosales et al. | 2020 | Australia &  New Zealand | 1980-2013 | Registry | Kidney | 17,628 | 60.8 | 45^‡^ | SMRs, CMR per 100,000 PY |
| Benoni et al. | 2020 | Sweden | 1992-2013 | Registry | All^1^ | 2,143^c^ | 65.0^c^ | N/A | HRs |
| Jeong et al. | 2020 | South Korea | 2003-2016 | Registry | Kidney | 9,915 | 60.2 | 44.7^d^ | SMRs |
| Jackson-Spence et al. | 2018 | UK | 2003-2014 | Registry | Kidney | 19,883 | 61.5 | 47.55 | SMRs |
| Acuna et al. | 2016 | Canada | 1991-2010 | Registry | All^2^ | 11,061 | 63.8 | 49.0^‡^ | SMRs |
| Friman et al. | 2022 | Finland | 1987-2016 | Registry | All^3^ | 6,548 | 62.0 | 49.6^‡^ | SMRs, CMR per 100 000 PY |
| Mazuecos et al. | 2009 | Spain | 1984-2007 | Registry | Kidney | 5,599 | N/A | N/A | Crude numbers of cancer deaths |
| Minguito-Carazo et al. | 2021 | Spain | 1984-2017 | Registry | Heart | 5,865 | 81.6 | 52.4 | CMR per 1000 PY |
| Miyazaki et al. | 2016 | Japan | 2001-2010 | Multi-centre (7) | Lung | 179 | 44.4^c^ | 43.0^‡ c^ | Crude number of cancer deaths |
| Galve et al. | 1999 | Spain | 1984-1996 | Multi-centre (10) | Liver | 1,827 | 72.2^c^ | N/A | Crude number of cancer deaths |
| Wang et al. | 2023 | USA | 1987-2018 | Registry | All^4^ | 671 127 | 62.0 | 50^‡^ | SMRs, MRR, CMR |
| Cheung et al. | 2012 | Hong Kong | 1972-2011 | Registry | Kidney | 4,674 | 58.6 | 43.7 | SMRs, CMR per 100,000 PY |

**Table S3 - Summary of included study characteristics**

***Notes***

*All^1^- Kidney, liver, heart, lung, pancreas, bowel transplants or a combination.*

*All^2^ – Kidney, liver, heart, lung, combined pancreas-kidney and “other” transplants.*

*All^3^ - Kidney, liver, heart, lung, and combined pancreas-kidney transplants.*

*All^4^ – Kidney, liver, heart and or lung, other/multiple transplants*

*^a^ Cohort type represents registry or multi-centre. (N) denotes the number of centres if multi-centre*

*^b^Age at transplantation; mean unless marked with ^‡^ (median)*

*^c^ Only reported recipients with cancer*

*^d^ Estimated using available data*

***Abbreviations***

*N/A = Not available, HRs = Hazard ratios, SMRs = Standardised mortality ratios, CMR = Crude mortality rate, PY = Person years, MRR = Mortality Rate Ratio*

**Table S4: Newcastle-Ottawa Scale scores for all studies**

| **Authors** | **Year** | **Representativeness  of exposed cohort** | **Selection of non-exposed  cohort** | **Ascertainment  of exposure** | **Demonstration that outcomes of interest  wasn't present at start of study** | **Comparability of cohorts on the basis of the design or analysis** | **Assessment of  outcome** | **Was follow-up long enough  for outcome to occur?** | **Adequacy of follow  up of cohorts** | **Total** |
| --- | --- | --- | --- | --- | --- | --- | --- | --- | --- | --- |
| Murray et al. | 2020 | 1 star | 1 star | 1 star | 1 star | 1 star | 1 star | 1 star | 0 stars | 7 stars |
| Taborelli et al. | 2024 | 1 star | 1 star | 1 star | 1 star | 1 star | 1 star | 1 star | 0 stars | 7 stars |
| Shalaby et al. | 2021 | 1 star | 1 star | 1 star | 1 star | 1 star | 1 star | 1 star | 0 stars | 7 stars |
| Yoon et al. | 2024 | 1 star | 1 star | 1 star | 1 star | 1 star | 1 star | 1 star | 0 stars | 7 stars |
| Imamura et al. | 2021 | 1 star | 1 star | 1 star | 1 star | 1 star | 1 star | 1 star | 0 stars | 7 stars |
| Rosales et al. | 2020 | 1 star | 1 star | 1 star | 1 star | 1 star | 1 star | 1 star | 0 stars | 7 stars |
| Benoni et al. | 2020 | 1 star | 1 star | 1 star | 1 star | 1 star | 1 star | 1 star | 0 stars | 7 stars |
| Jeong et al. | 2020 | 1 star | 1 star | 1 star | 1 star | 1 star | 1 star | 1 star | 1 star | 8 stars |
| Jackson-Spence et al. | 2018 | 1 star | 1 star | 1 star | 0 star | 2 stars | 1 star | 1 star | 0 stars | 7 stars |
| Acuna et al. | 2016 | 1 star | 1 star | 1 star | 1 star | 2 stars | 1 star | 1 star | 0 stars | 7 stars |
| Friman et al. | 2022 | 1 star | 1 star | 1 star | 1 star | 2 stars | 1 star | 1 star | 1 star | 9 stars |
| Mazuecos et al. | 2009 | 1 star | 1 star | 1 star | 1 star | 1 star | 1 star | 1 star | 0 stars | 7 stars |
| Minguito-Carazo et al. | 2021 | 1 star | 1 star | 1 star | 1 star | 1 star | 1 star | 1 star | 0 stars | 7 stars |
| Miyazaki et al. | 2016 | 1 star | 1 star | 1 star | 1 star | 1 star | 1 star | 1 star | 0 stars | 7 stars |
| Galve et al. | 1999 | 1 star | 1 star | 1 star | 1 star | 1 star | 1 star | 1 star | 0 stars | 7 stars |
| Wang et al. | 2023 | 1 star | 1 star | 1 star | 1 star | 2 stars | 1 star | 1 star | 0 stars | 8 stars |
| Cheung et al. | 2012 | 1 star | 1 star | 1 star | 1 star | 1 star | 1 star | 1 star | 0 stars | 7 stars |


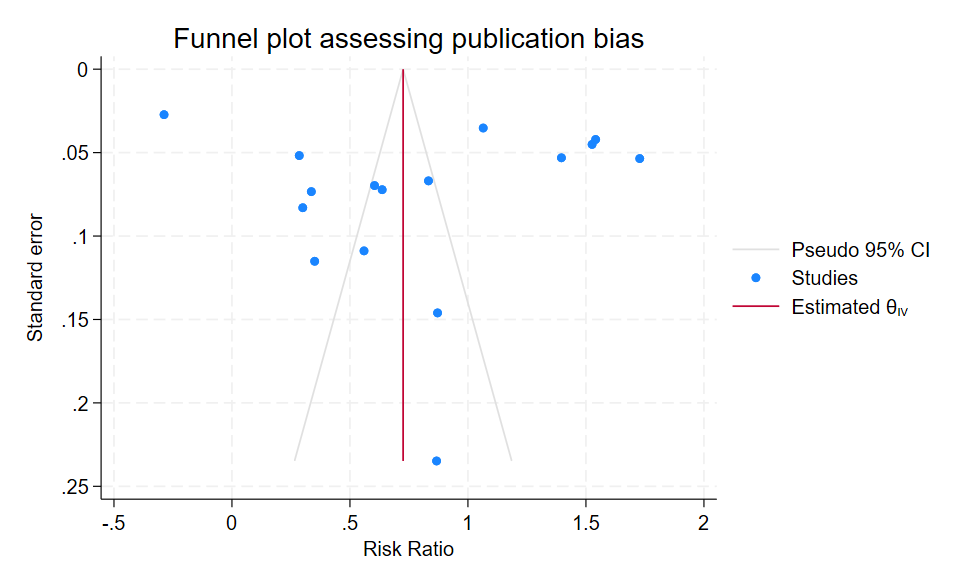


**FigureS1 - Funnel plot assessing publication bias of included studies**

| **Cancer site** | **Pooled SMRs  (95% CI)** | **I² (%)** |
| --- | --- | --- |
| Colorectal | 1.94 (1.26-2.99) | 84.39 |
| Lung | 1.50 (1.06-2.13) | 89.11 |
| Breast | 1.54 (1.14-2.07) | 37.79 |
| Prostate | 1.09 (0.71-1.69) | 73.45 |
| Non-Hodgkin Lymphoma | 7.62 (3.30-15.59) | 97.8 |

**Table S5: Pooled SMRs for site specific cancers among kidney transplant recipients**
